# Optimization of the Rheological Properties of Fat Replacers Based on Inulin at Different Degrees of Polymerization and Their Application in Beef Burgers

**DOI:** 10.3390/foods14122127

**Published:** 2025-06-18

**Authors:** Michela Pia Totaro, Mariana Miccolis, Davide De Angelis, Giuseppe Natrella, Francesco Caponio, Carmine Summo, Michele Faccia

**Affiliations:** Department of the Soil, Plant and Food Science DiSSPA, University of Bari Aldo Moro, Via Amendola, 165/a, 70126 Bari, Italy; michela.totaro@uniba.it (M.P.T.); mariana.miccolis@uniba.it (M.M.); giuseppe.natrella@uniba.it (G.N.); francesco.caponio@uniba.it (F.C.); carmine.summo@uniba.it (C.S.); michele.faccia@uniba.it (M.F.)

**Keywords:** fat replacement, inulin, mixture design, rheological properties, beef burgers, degree of polymerization

## Abstract

Fats play a key role in the rheological and textural properties of meat products. However, growing awareness of the link between diet and disease has stimulated research on fat replacers that can replicate these functional properties. Inulin, a β-D-fructose polymer available in various degrees of polymerization (DP), is promising as a fat replacer due to its gel-forming ability in aqueous systems and its neutral sensory profile. This study focused on optimizing the formulation of inulin gel-based fat replacers for producing reduced-fat beef burgers. A D-optimal mixture-process design was employed, considering inulin with high-DP (HDP) and low-DP (LDP). The aim was to determine the optimal amount of inulin, water, and guar gum to achieve gels with rheological properties (η, shear viscosity; K, consistency index) similar to beef fat. The optimal formulations consisted of 51.52% inulin, 48.48% water, 1.50% guar gum for LDP gel, and 39.12% inulin, 60.88% water, 1.50% guar gum for HDP gel. These gels demonstrated shear viscosity and consistency indices comparable to beef fat. While rheological behavior at constant temperatures was similar, inulin gels showed increasing viscoelastic moduli (G′ and G″) with temperature, in contrast to the melting behavior of animal fat. When used in beef burger formulations, the optimized gels resulted in improved cooking yields, reduced shrinkage, and better dimensional stability compared to conventional controls. These benefits are attributed to the hydrophilic and stabilizing properties of inulin. The findings support the use of inulin-based gels as effective fat replacers, offering a promising strategy to reduce fat content in meat products without compromising functional quality.

## 1. Introduction

Growing consumer awareness regarding diet and health has increased the demand for foods with enhanced nutritional properties, including low-fat meat products [1]. This is motivated by the fact that traditional meat products are often rich in saturated fatty acids (SFA) and cholesterol, which have been linked to an increased risk of cardiovascular diseases, obesity, and certain cancers, such as breast, colon, and prostate cancer [2]. In response, the World Health Organization (WHO) recommends that dietary fat should contribute to 15–30% of a daily caloric intake, with saturated fat limited to 10% of total calories, while cholesterol intake should not exceed 300 mg per day [3]. Despite its negative health implications, animal fat determines the rheological, textural, physicochemical, and sensory properties of meat products [4]. Consequently, developing low-fat meat products that maintain desirable quality characteristics remains a significant challenge for both researchers and food companies [2]. Dietary fibers can be incorporated into meat formulations to substitute fat, as they can enhance moisture retention and mimic fat-like properties [5]. Among these, inulin, a linear polysaccharide composed of β (2-1)-linked fructose units [6], has been widely explored as a fat replacer due to its functional properties. Indeed, it contributes to improve yield, texture, and overall quality of meat formulations [1,7], while also offering health benefits such as reduced risks of coronary heart disease, diabetes, irritable bowel syndrome, and obesity [8]. The technological functionality of inulin largely depends on its degree of polymerization (DP), which ranges from 2 to 60 units and directly influences its physicochemical properties and prebiotic activity [9]. Specifically, a high degree of polymerization (HDP) inulin (DP ≥ 23) exhibits superior gelling properties and higher viscosity compared to a low degree of polymerization (LDP) inulin (DP ≤ 10). HDP inulin is typically used as a fat replacer, providing a fat-like mouthfeel, whereas LDP inulin, being sweeter, can partially substitute for sucrose’s flavor [9]. The literature reviews indicate that HDP inulin has been most extensively investigated in meat products such as burgers [7,10], sausages [4,11], ham [12], and patties [13]. Conversely, although LDP inulin has been less studied, Keenan et al. [14] reported that it reduced cooking losses in low-fat sausages, while Felisberto et al. [15] found improvements in tenderness and sensory acceptance in Bologna-type sausages. In these previous studies, inulin was predominantly used as a powder directly incorporated into the meat matrix.

When developing and optimizing an inulin-based gel as a fat replacer, the influence of the inulin ratios and types on the structural properties should be carefully considered [8]. To achieve this goal, a Design of Experiments (DoE) approach can be used [16]. In the present study, a D-optimal mixture × process design was developed to optimize the formulation of HDP and LDP inulin gels, combined with water and guar gum, to achieve rheological properties—shear viscosity (η) and consistency index (K)—comparable to those of beef fat. Then, two optimal formulations were used to partially substitute the beef fat in burgers, evaluating the cooking properties of low-fat beef burgers (5% back fat) and comparing them to conventional beef burgers (12% back fat).

## 2. Materials and Methods

### 2.1. Materials

Low-degree polymerization (LDP, DP ~10, Fibruline^®^ Instant, chicory root fiber powder; 92% inulin on a dry matter basis, 88% dietary fiber, 5% moisture, 0.3% ash) and high-degree polymerization (HDP, DP > 20, Fibruline^®^ XL Chicory Root Fiber-Powder, ~99.5% inulin on dry matter, 94.5% dietary fiber, 4% moisture, 0.2% ash) inulin were obtained from Cosucra (Warcoing Industrie, Brussels, Belgium). Guar gum was sourced from Reire (Reggio Emilia, Italy). Distilled water was generated using an Arium^®^ 611 UV system (Sartorius, Göttingen, Germany). Lean beef and beef back fat were kindly provided by Matarrese S.r.l. (Alberobello, Italy), where the production of burgers and cooking processes took place.

### 2.2. Experimental Design and Inulin-Based Gels Preparation

The optimization of the fat replacer formulations was carried out using an approach based on the Design of Experiments. Specifically, considering that inulin and water were the main components, their combinations were defined using a D-Optimal mixture design for two dependent components according to the D-Optimal criterion and a quadratic model. Additionally, since guar gum is a minor component, used in a much smaller amount compared to inulin and water, it was considered as an independent variable [17]. Consequently, for each combination of water and inulin, three levels of guar gum concentration were considered. The details of each experiment are reported in Table 1, and the concentrations were chosen according to a previous study [18] and preliminary trials. Moreover, three replicate points were also included in each model to consider the variability related to the preparation process [19].

Inulin-based gels were prepared following the method described by De Souza Paglarini et al. [20], with modifications. Specifically, inulin (LDP and HDP) and water were homogenized using an Ultra-Turrax T-18 (IKA-Werke GmbH & Co. KG, Staufen, Germany) at 10,000 rpm for 30 s. Guar gum was then added, and the mixture was further homogenized at 10,000 rpm for 1 min. The resulting gels were immediately analyzed.

### 2.3. Rheological Determination

The rheological properties of inulin-based gels and beef back fat were measured with a rheometer (HAAKE MARS iQ Air, Thermo Fisher Scientific, Waltham, MA, USA) equipped with a parallel plate geometry (P35/Ti-02180932). All the determinations were carried out in triplicate.

A shear ramp test was carried out to measure the viscosity of the gels, and the analysis was conducted using a 0.8 mm gap between the plates at 25 °C, by increasing the shear rate from 0 to 100 s^−1^, as reported by De Angelis et al. [21] with minor modification. The viscosity data (η, expressed in Pa s) were fitted to the Ostwald–de Waele model, according to the following equation:

η = K × *ɣ̇* ^n−1^

where η = viscosity (Pa s), K = consistency index (Pa s^n−1^), *ɣ̇* = shear rate (s^−1^), and n = flow behavior index.

The frequency-dependent behavior of gels was determined using a 0.8 mm gap between the plates by an oscillatory frequency sweep at 25 °C, carried out as described by Krystyjan et al. [22], with minor modifications. The frequency varied from 0.1 to 10 Hz at 1% strain (which is within the linear viscoelastic regime). The elastic modulus (G′) and viscous modulus (G″) were evaluated as a function of the frequency.

The temperature sweep analysis was conducted according to Hu et al. [23], with some modification. The gels were analyzed at 1 Hz frequency, 0.1% strain (within the linear viscoelastic regime), and with a 2 mm gap between plates. The temperature varied from 25 to 70 °C at a heating rate of 5 °C min^−1^.

### 2.4. Selection of the Optimal Formulation of Inulin-Based Gels

A graphical optimization of the formulations was performed using Design-Expert 11 (StatEase Inc., Minneapolis, NM, USA) with the aim of achieving viscosity (η) and consistency index (K) similar to beef back fat. The optimal formulations were selected within the optimal areas of the experimental domain and were prepared as described in Section 2.2. To validate the model predictions, rheological evaluations of the gels were conducted as described in Section 2.3. The measured data were compared to the predicted values, and if they fell within the 95% confidence intervals of the predicted values, they were considered as acceptable.

### 2.5. Preparation of Beef Burgers Containing Optimal Formulation of Inulin-Based Gels

The optimized LDP and HDP gels were used to produce low-fat beef burgers (5% beef back fat) compared to conventional beef burgers (12% beef back fat, which is similar to the common traditional beef burger generally commercialized in the Italian market). Beef burgers were produced and cooked at Matarrese S.r.l. (Alberobello, Italy). The pieces of meat were previously cleaned to remove visible fat, cut into strips, and ground with a refrigerated TC 22-32 NEVADA meat grinder (Sirman, Padua, Italy) equipped with 4.5 mm disks. Beef back fat was also similarly ground on a 4.5 mm disk. Three different trials were prepared according to the formulations reported in Table 2 and two different batches were prepared for each thesis.

The fat content in the low-fat formulations was determined based on previous research. Low-fat meat products should not exceed 10% fat, while very low-fat products typically contain around 5% fat [24,25]. Moreover, maintaining fat levels above this threshold is essential to mask certain flavors associated with lean meat [26]. After grinding, the ingredients were added to the lean beef meat, and mixed for 5 min using a commercial mixer (IP 10–20 M, Sirman, Padua, Italy) to create a uniform burger paste. No additional ingredients were included in the formulations. Burgers with constant weight (100 g) were shaped using a round plate mold (1 cm thick × 10 cm diameter). Cooking was performed using a gas cooker (NEBG92G_900 line, Silko Ali Group, Vittorio Veneto, Italy) at 350 °C for 4 min following the American Meat Science Association methodology (AMSA, 2015) guidelines [27].

### 2.6. Cooking Parameters of Beef Burgers

The following determinations were performed on the cooked burgers to estimate the cooking loss, cooking yield and shrinkage, as reported in Heydari et al. [28]:

$$Cooking\;loss\%=\left(\frac{raw\;weight-cooked\;weight}{raw\;weight}\right)\times 100$$

$$Cooking\;yield\%=\left(\frac{cooked\;weight}{raw\;weight}\right)\times 100$$

$$Shrinkage\%=\frac{\left(raw\;thickness-cooked\;thickness\right)+(raw\;diameter-cooked\;diameter)}{\left(raw\;thickness+raw\;diameter\right)}$$

The reduction in diameter and increase in thickness of beef burger were determined by analyzing the dimensions of the burgers both before (raw) and after cooking using the following equations:

$$Diameter\;reduction\%=\left(\frac{raw\;diameter-cooked\;diameter}{raw\;diameter}\right)\times 100$$

$$Thickness\;increase\%=\left(\frac{cooked\;thickness-raw\;thickness}{raw\;thickness}\right)\times 100$$

### 2.7. Statistical Analysis

The responses of the experimental design were modelled according to the postulated quadratic × linear model and the following equation:

*y* = *b*_1_*X*_1_ + *b*_2_*X*_2_ + *b*_12_*X*_1_*X*_2_ + *b*_13_*X*_1_*X*_3_ + *b*_23_*X*_2_*X*_3_ + *b*_123_
*X*_1_*X*_2_*X*_3_

Specifically, *y* indicates the response, *b*_1_ and *b*_2_ indicate the coefficients of the linear terms, *b*_12_, *b*_13_, and *b*_23_ indicate the coefficients of the two-way interaction and *b*_123_ indicates the three-way interaction. *X*_1_, *X*_2_, and *X*_3_ are the components under investigation, i.e., inulin, water, and guar gum, respectively. The coefficient of determination (R^2^), the adjusted coefficients of determination (R^2^ adj), as well as their significance (*p* ≤ 0.05) were calculated by the software Design-Expert 11 (StatEase Inc., Minneapolis, NM, USA). Data were subjected to One-Way Analysis of Variance (ANOVA) followed by Tukey’s HSD (Honestly Significant Differences) test for multiple comparisons at a significance level *p* = 0.05. The differences between the experimental and the control were evaluated using Dunnett’s multiple comparisons test at *p* = 0.05. All data were processed by Minitab Statistical Software 19.1 (Minitab Inc., State College, PA, USA). The plots of the rheological data properties were made using GraphPad Prism version 9 (GraphPad Software, San Diego, CA, USA).

## 3. Results and Discussion

### 3.1. Models Evaluation

The coefficients of the regression models calculated for the responses, and their coefficients of determination and significance, are reported in Table 3. The viscosity (η) and consistency index (K) were chosen as optimization parameters, as these are essential to identify formulations of inulin-based gels with different degrees of polymerization with flow and consistency properties similar to those of beef fat. Shear viscosity reflects the resistance of material to flow under shear stress, while the consistency index indicates its structural integrity and stability [29,30]. The shear rate of 10 s^−1^ was considered because the mixing processing occurs in a wide range of shear rate, including 10 s^−1^ [29].

The rheological properties of the inulin-based gels, specifically their consistency index (K) and apparent viscosity (η, at 10 s^−1^), were significantly influenced by the composition of inulin, water, and guar gum. The statistical models developed for both LDP and HDP inulin gels were found to be highly predictive, as indicated by the high coefficients of determination and low *p*-values. This confirms that the experimental design effectively captured the interactions between the ingredients and their impact on gel structure.

**Figure 1:**
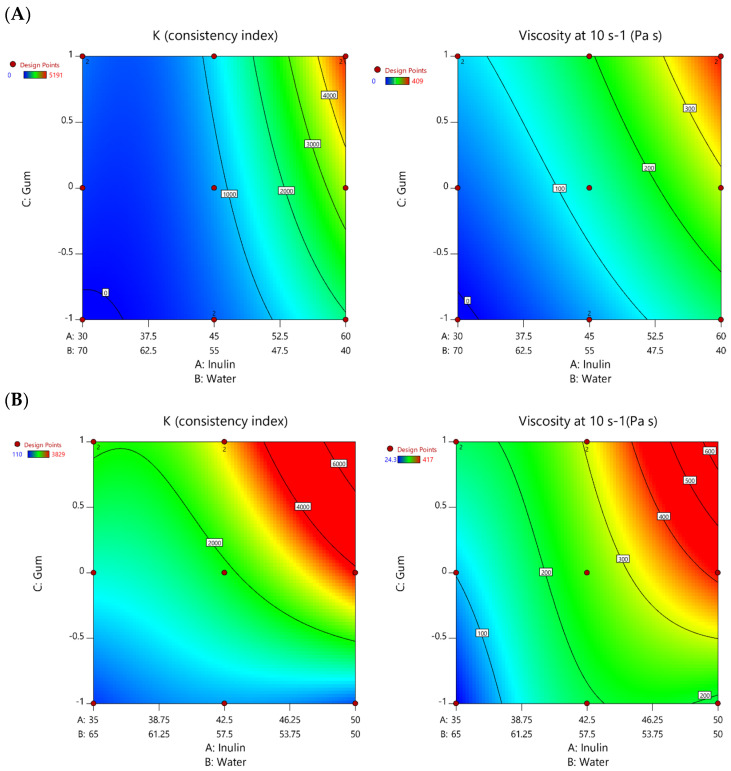
Contour plots depicting the variations in the consistency index (K) and apparent viscosity (η, Pa s at *ɣ̇* 10 s^−1^) of LDP (**A**) and HDP (**B**) gels. Color variation from blue to red indicates an increase in the considered parameter.

The linear coefficients were always significant for LDP, while for HDP gels, linear coefficients were significant only for the viscosity. According to the magnitude of the coefficients, it seems that the strongest effect on the rheological properties is given by the inulin content as well as by its interaction with the guar gum (Table 3). Inulin is known to act as a structuring agent by forming a gel network in water, which explains why increasing its concentration led to higher viscosity and consistency. However, the responses varied between LDP and HDP gels due to differences in molecular size and gelling behavior [31,32]. The interaction effects also significantly contributed to the observed behavior. For example, the negative coefficient for the inulin–water interaction indicates that increasing the water content weakened the gel structure, likely due to dilution effects. However, this effect was more pronounced in LDP gels, suggesting that shorter-chain inulin is more susceptible to water-induced weakening than longer-chain inulin [6,32]. Strong positive effects were observed for the inulin–gum interaction, particularly in HDP gels, indicating that guar gum synergically enhances the structuring ability of inulin [9,33]. To better understand and visualize the overall effect of the components on the responses under investigation, the contour plots of the most relevant rheological properties should be examined [19], and they are depicted in Figure 1.

By analyzing these plots, it is evident that viscosity and gel consistency can be adjusted through different combinations of the three factors, providing a certain degree of flexibility. For example, the green areas of the contour plots, which represent an intermediate range of viscosity and consistency index within our experimental domain, can be achieved at either higher or lower inulin-to-water ratios by varying guar gum concentration. Conversely, reducing inulin and increasing guar gum can produce a similar structure, which may be beneficial in applications where excessive inulin content could affect sensory properties [2,34,35].

This suggests that gel structure can be tailored for specific food applications based on additional parameters, like, for example, the desired final moisture content of the products or its fiber content.

### 3.2. Rheological Properties of Inulin-Based Gels and Back Fat and Optimization of Formulations

The results of the consistency index and viscosity of the inulin-based gels and beef back fat are reported in Table 4. As can be observed, the rheological properties of the inulin-based gels can be tuned to match the consistency index (K) and apparent viscosity (η) of beef back fat. This is particularly important in meat products where texture and mouthfeel are strongly influenced by fat content [36,37]. Based on these findings, graphical optimization was carried out to select two optimal formulations (one for LDP and one for HDP inulin gels).

**Figure 2:**
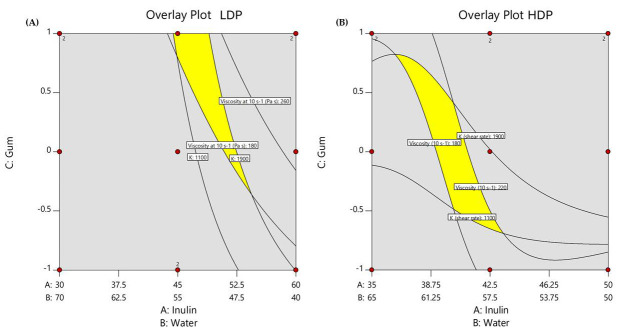
Overlay plots showing the location (yellow zone) of optimized LDP (**A**) and HDP (**B**) inulin gel-based formulations.

The graphical optimization approach was chosen due to the limited number of variables involved in this study, aligning with recommendations from previous research [38]. This method allowed for the identification of formulations that best matched the rheological properties of beef back fat by overlaying the contour plots of the consistency index (K) and apparent viscosity (η) (Figure 1). The resulting overlay plots are depicted in Figure 2, and two formulations, one for HDP and one for LDP, were chosen according to the best solutions in terms of ratio of ingredients.

The final optimized formulations are presented in Table 5, together with the results of the analysis of the consistency index and viscosity of the gels. The results highlight that no significant differences were found among the optimized formulation and the beef back fat, confirming that both HDP and LDP gels can be tailored to match the rheological properties of beef fat, making them suitable fat substitutes. Moreover, the results also validate the model predictions. The observed values fell within the 95% prediction interval of the models, demonstrating the efficiency and reliability of the Design of Experiment approach used in the optimization of both the ingredients. This is particularly relevant, as experimental models are often not validated [39], although validation is essential to assess the real applicability of the developed models.

### 3.3. Rheological Properties of the Optimized Formulations and Comparison with the Beef Back Fat

**Figure 3:**
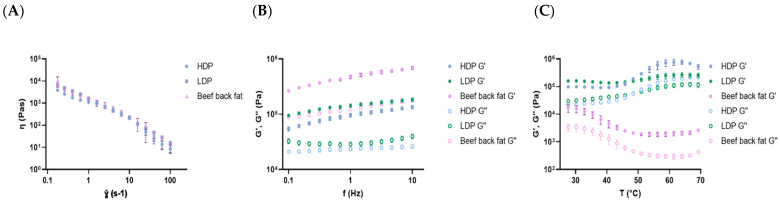
Rheological properties of the optimized formulations and the beef back fat: (**A**) shear rate ramp test, (**B**) frequency sweep analysis, (**C**) temperature sweep analysis.

The rheological properties of beef back fat and optimized formulations of inulin gels based are shown in Figure 3. The analysis of the viscosity carried out by a shear ramp test highlighted the successful optimization study of the gels with respect to the beef back fat. In fact, the curves are nearly overlapped, indicating that inulin-based gels and beef fat had similar viscous properties. This finding is important for the development of fat replacers, as data from shear-dependent behaviors are related to sensory viscosities, thickness, and smoothness [40].

As shown in Figure 3A, viscosity decreased with an increasing shear rate, indicating a typical shear-thinning behavior of pseudoplastic non-Newtonian fluids [41]. This rheological behavior is characteristic of long-chain polymers [42], including triglycerides in beef back fat [43] and has also been observed in inulin-based suspensions [9,44]. As regards the studied fat replacers, the re-arrangement of inulin molecules in the direction of flow, as well as the weakening of the intermolecular force promoted by the shear, could explain the reduction in viscosity [45,46]. When semisolid and liquid foods are eaten, the shear-thinning behavior helps to create a favorable mouthfeel [47]. Therefore, as both beef back fat and fat replacers exhibited a shear-thinning behavior, inulin gels could be successfully applied to mimic fat properties, in low-fat burgers. Moreover, the shear-thinning properties of inulin gels provide significant functional advantages in food processing and meat product preparation, in terms of enhanced processability, energy efficiency, and improved textural quality in reduced-fat formulations [44]. As suggested by Li et al. [44], the decrease in viscosity at high shear rates enhances the dispersion and integration of inulin-based fat replacers during kneading, thereby improving overall processability and contributing to a more uniform texture and better structural integrity in the final product. Specifically, as the gels become less viscous under shear stress, they integrate more easily into lean meat matrices, reducing the need for excessive mechanical force during mixing. This property can help minimize over-processing, which is crucial for maintaining the integrity of muscle fibers and preserving the textural attributes of the final product [48]. However, to fully understand how these gels behave under real processing conditions, future studies should incorporate large strain amplitude tests, which are more representative of industrial-scale operations [49].

The frequency-dependent behavior of the gels is shown in Figure 3B. Within the frequency range under investigation, G′ was consistently higher than G″, indicating a dominant elastic response. Moreover, G′ exhibited an increasing trend at higher frequencies, suggesting the presence of a gel network primarily stabilized by non-covalent interactions [50]. No evidence of crossover between G′ and G″ was observed, suggesting that the network remained stable over time [51]. The G’ of beef fat increased slightly more than that of inulin gels, implying that it became more elastic at a microscopic level as the frequency increased. Overall, despite the higher absolute values of both moduli, beef fat exhibited a viscoelastic behavior similar to that of inulin gels. The optimized LDP gel had slightly higher dynamic moduli than the HDP one. This could be reasonably due to the higher inulin content in LDP gel, compared to the HDP one (Table 5), and it may be attributed to specific functional properties of LDP inulin, such as its higher solubility [44]. Furthermore, the greater chain flexibility associated with short-chain inulin [32] may enhance its interaction with guar gum, contributing to a more cohesive and dense gel matrix [52]. Our results are in line with those of other authors [18,41]. Therefore, these findings suggest that LDP inulin, when properly formulated, can perform similarly to HDP inulin in structuring applications, offering more flexibility in meat product reformulation, particularly in products aiming to increase dietary fiber content without compromising texture.

Figure 3C shows the thermal behavior of beef back fat and optimized gels in terms of the elastic modulus (G′) and viscous modulus (G″), as a function of the temperature (from 25 to 70 °C). Animal fat and inulin gels exhibited G’ values higher than G″, throughout the temperature range studied (Figure 3C), indicating a viscoelastic behavior. Nevertheless, dynamic moduli of the fat replacers were higher than those of beef back fat. Indeed, the primary difference between inulin-based gels and beef back fat became evident when subjected to a temperature ramp. Inulin gels did not exhibit any decrease in storage and loss moduli as the temperature increased, highlighting their thermal stability. Conversely, for beef fat, a decreasing trend in G′ and G″ values during heating was observed, which is consistent with its melting, as already reported by other authors [53,54,]. This melting phenomenon could explain cooking losses of meat products [5,54]. In agreement with Zhang et al. [55], during the initial phase of heating, the rise in G’ and G″ for fat replacers was relatively modest. However, as the temperature rose to 40 ° C–50 ° C, the G′ and G″ values showed an increase, greater for HDP gel, suggesting that inulin of higher DP promotes more entanglement in the gel [56]. The increase in G′ value with the continuous rise in temperature could be caused by the thickening effect of the guar gum used in the formulations, which limits the mobility of fat replacers [57]. Contrary to the optimized LDP gel, with the increase in temperature, G′ value of HDP gel increased rapidly until the maximum and then exhibited a slight decrease with the high temperature. The latter result indicates the destruction of the gel structure with prolonged heating [9,55]. This thermal behavior may be advantageous during cooking, as the thermal stability of inulin gels contributes to improved moisture retention and dimensional stability. Consequently, the resistance of these gels to thermal breakdown could help to prevent excessive water loss, enhancing cooking yield and reducing deformation or shrinkage in processed meat products subjected to high-temperature treatments such as grilling or baking, as is further illustrated in Section 3.4.

Finally, to better explain the behavior and performance of the inulin-based gels, future studies should also focus on their microstructural characterization using scanning electron microscopy (SEM). In fact, the microstructural arrangement of the gel may significantly influence key sensory attributes, such as the mouthfeel of the final product [52]. Moreover, morphological characteristics can help to explain the functional properties of gelled fat replacers, including solubility, gelling capacity, interaction with other ingredients, and thermal and structural stability in various food matrices [58].

### 3.4. Cooking Properties of Cooked Beef Burgers

Table 6 shows the results of the statistical analysis of key cooking parameters for reduced-fat beef burgers formulated with inulin gels (LDP and HDP) compared to the control. These parameters were evaluated to determine the impact of fat substitution on the technological properties of the burgers, during cooking. The inclusion of inulin, known for its water-holding capacity, can significantly influence factors, such as cooking loss and final yield compared to traditional beef burgers, containing animal fat [34]. Assessing these parameters ensures that reduced-fat formulations retain similar quality attributes to conventional burgers, minimizing moisture loss, while preserving juiciness and texture. Indeed, preventing excessive dimensional changes during cooking is a key factor in maintaining consumer-perceived quality [59].

Cooking loss, which refers to weight reduction due to moisture and fat loss during cooking [60], was significantly higher (*p* < 0.05) in the CTR compared to the reduced-fat formulations. As a consequence, the addition of inulin gels led to a notable improvement in cooking yield, with both LDP-B and HDP-B exhibiting significantly higher values than the control (*p* < 0.05). This enhancement is likely due to the presence of hydrophilic groups and the hygroscopic nature of inulin which may contribute to increased moisture retention in the burgers [34]. Moreover, the fat melting phenomena observed in the temperature sweep analysis could have also contributed to lower cooking yield in CTR. Thus, the rheological analysis of the thermal behavior of inulin gels (Section 3.3), likely contributes to these improvements by maintaining gel structure during cooking, thereby preventing the typical melting seen in beef back fat. The decrease in the dynamic moduli of beef fat upon heating reflects melting and phase changes that lead to moisture loss, which negatively affects cooking yield and textural integrity [5,54]. By contrast, the observed behavior of inulin-based gels helps to preserve the gel network, which acts as a physical barrier that reduces migration of moisture and fat dripping during heat exposure [55].

By comparing LDP and HDP burgers, HDP-B demonstrated a significantly lower cooking loss and higher cooking yield compared to LDP-B. In agreement with what was observed in the temperature sweep analysis, this can be attributed to the more extensive gel network in HDP inulin, which enhances water retention and reduces moisture loss [32,61].

At the same time, the denaturation of meat protein linked to the loss of water and fat, is the main cause of diameter reduction and of the consequent increase in thickness in meat products [10]. CTR exhibited the most significant dimensional changes, with the highest values for both diameter reduction and thickness increase. Conversely, the inulin-based formulations showed significantly lower changes (*p* < 0.05), likely due to the stabilizing properties of inulin, which help maintain meat structure and cohesion [5]. At the same time, shrinkage, defined as the overall reduction in size or volume during cooking [62], followed a similar trend. CTR exhibited the highest value of shrinkage, which can be attributed to the lack of the stabilizing effect of inulin. By contrast, both inulin-based formulations exhibited lower shrinkage values, with HDP-B showing a significantly lower value compared to CTR, likely due to its higher ability to face higher cooking temperatures while maintaining its structure. This aligns with previous research confirming the heat-resistant properties of inulin compared to beef fat [14,34]. These findings highlight the potential of inulin as a functional ingredient in reduced-fat beef burgers. By improving moisture retention, reducing cooking loss, enhancing yield, and maintaining dimensional stability, inulin-based formulations offer a promising approach to developing healthier meat products without compromising quality.

However, as a future perspective, the sensory properties of the gels and of the low-fat burgers should be considered. Moreover, future research should include sensory trials of burgers made with inulin gels to better understand their impact on consumer perception.

## 4. Conclusions

This study demonstrates that inulin-based fat replacers can be effectively optimized to replicate the shear viscosity and consistency of beef back fat. Optimal formulations were identified using a D-optimal mixture × process design, with separate models for high- and low-degree polymerization inulin. In general, LDP inulin gels required higher inulin concentrations to achieve rheological properties, such as viscosity and consistency, comparable to those of HDP gels. The results indicated that desired viscosity and consistency values can be achieved through different combinations of inulin, water, and guar gum. This tailors the gel properties for specific food applications, such as adjusting moisture or fiber content, while minimizing the risk of adverse sensory properties from excessive inulin use. The optimized inulin gels exhibited viscous and viscoelastic behavior similar to beef back fat. However, significant differences emerged under temperature ramp conditions. In fact, inulin gels showed increasing values of G′ and G″ with rising temperature, while beef back fat exhibited a decreasing trend due to melting phenomena. These findings suggest that further formulation adjustments are needed to better replicate the thermal behavior of animal fat.

Interestingly, cooking properties showed that reduced-fat burgers containing the optimized gels had an improved cooking yield, reduced shrinkage, and had better dimensional stability compared to conventional burgers. These results can be attributed to the hydrophilic and stabilizing properties of inulin, which enhance moisture retention and structural integrity during thermal processing.

Overall, these findings support the potential of inulin-based gels as effective fat replacers in meat products. Future research should focus on consumer acceptance and nutritional and sensory aspects to have a more comprehensive basis for further exploitation in the meat industry.

## Figures and Tables

**Table 1:** Formulation of the fat replacers according to the experimental design.

Trial	Inulin (*X*1) (g/100 g)	Water (*X*2) (g/100 g)	Guar Gum (*X*3) (g/100 g)	Trial	Inulin (*X*1) (g/100 g)	Water (*X*2) (g/100 g)	Guar Gum (*X*3) (g/100 g)
LDP Gel				HDP Gel			
1	30	70	0.5	1	35	65	0.5
2	30	70	1.5	2	35	65	1.5
3	30	70	3.5	3	35	65	3.5
3 *	30	70	3.5	3 *	35	65	3.5
4	45	55	0.5	4	42.5	57.5	0.5
4 *	45	55	0.5	5	42.5	57.5	1.5
5	45	55	1.5	6	42.5	57.5	3.5
6	45	55	3.5	6 *	42.5	57.5	3.5
7	60	40	0.5	7	50	50	0.5
8	60	40	1.5	8	50	50	1.5
9	60	40	3.5	9	50	50	3.5
9 *	60	40	3.5	9 *	50	50	3.5

* Replicates. Abbreviations: LDP, low degree of polymerization; HDP, high degree of polymerization.

**Table 2:** Formulations of beef burgers.

Sample	Lean Meat (g/100 g)	Beef Back Fat (g/100 g)	LDP Gel (g/100 g)	HDP Gel (g/100 g)
CTR	88	12	-	-
LDP-B	88	5	7	-
HDP-B	88	5	-	7

Abbreviations: CTR, control burger; LDP-B, low-fat beef burger formulated with LDP gel; HDP-B, low-fat beef burger formulated with HDP gel.

**Table 3:** Regression coefficients of the model and their significance for K (consistency index, Pa s^n-1^) and η (apparent viscosity, Pa s at *ɣ̇* 10 s^−1^) determined on the LDP and HDP gels produced by the experimental design (A: inulin, B: water, C: guar gum).

Response	A (Inulin)	B (Water)	AB (Inulin–Water)	AC (Inulin–Gum)	BC (Water–Gum)	ABC	R^2^	R^2^_adj_	*p*-Value
LDP gel									
K (Pa s^n−1^)	**3510**	**251**	**−4208**	**1600**	**325**	**−2428**	**0.998**	**0.996**	**<0.001**
η (Pa s at *ɣ̇* 10 s^−1^)	**280**	**37**	−124	**125**	**47**	**−81**	**0.986**	**0.974**	**<0.001**
HDP gel									
K (Pa s^n−1^)	3830	1202	−2559	**3491**	**911**	**−4035**	**0.965**	**0.913**	**0.006**
η (Pa s at *ɣ̇* 10 s^−1^)	**417**	**102**	−41	**232**	**81**	**−393**	**0.930a**	**0.982**	**0.020**

The significant effects are indicated in bold (*p* < 0.05).

**Table 4:** Consistency index and viscosity of the experimental trials and the beef back fat.

Trial	K (Pa s^n−1^)	η (Pa s at *ɣ̇* 10 s^−1^)
LDP 1	0 ± 0	0 ± 0
LDP 2	102.49 ± 3.83	17.09 ± 0.52
LDP 3	629.07 ± 28.98	90.71 ± 7.21
LDP 4	519.15 ± 37.72	73.75 ± 7.35
LDP 5	718.15 ± 76.86	85.27 ± 16.51
LDP 6	1239 ± 27.57	214.75 ± 0.77 *
LDP 7	1868.5 ± 96.87 *	153.9 ± 9.47
LDP 8	3594.5 ± 388.2	281.1 ± 56.28 *
LDP 9	5191 ± 43.84	409.3 ± 127.56
HDP 1	109 ± 0.30	24.28 ± 0.40
HDP 2	1565 ± 51.10 *	95.45 ± 8.05
HDP 3	1973 ± 231 *	181 ± 0.50 *
HDP 4	922 ± 22.90 *	190 ± 0.45 *
HDP 5	1398 ± 555 *	151 ± 17.5
HDP 6	3046 ± 825	327 ± 44
HDP 7	338.3 ± 57.2	185 ± 7.3 *
HDP 8	3830 ± 455	417 ± 91
HDP 9	n.d.	n.d.
Beef back fat	1525.33 ± 475.84	220.77 ± 40.43

* indicates no significant difference with the beef back fat according to Dunnett’s test (*p* > 0.05). n.d., not determined due to technical reasons.

**Table 5:** Results of K and η (Pa s at *ɣ̇* 10 s^−1^) of inulin gels optimal formulations.

Sample	Inulin (%)	Water (%)	Guar Gum (%)	K (Pa s^n−1^)	η (Pa s at *ɣ̇* 10 s^−1^)
LDP gel	51.52	48.48	1.50	1506.67 ± 15.28 a (1735 ± 115)	211.10 ± 21.55 a (186 ± 23)
HDP gel	39.12	60.88	1.50	1218.67 ± 101.52 a (1414 ± 368)	215.13 ± 28.29 a (180 ± 46)
Beef fat				1525.33 ± 475.84 a	220.77 ± 40.44 a

Mean ± standard deviation. The predicted values are reported in the brackets. Means from the same column followed by different letters indicate significant differences in the function of the treatment (*p* < 0.05) by one-way ANOVA followed by Tukey’s HSD test. Abbreviations: K, consistency index; η, apparent viscosity.

**Table 6:** Results of the statistical analysis (one-way ANOVA) (mean ± standard deviation) of cooking parameters.

Sample	Cooking Loss (%)	Cooking Yield (%)	Diameter Reduction (%)	Thickness Increases (%)	Shrinkage (%)
CTR	26.67 ± 1.25 a	73.33 ± 1.25 c	21.00 ± 0.25 a	7.50 ± 2.50 a	18.49 ± 0.45 a
LDP-B	24.09 ± 0.65 b	75.09 ± 0.65 b	17.50 ± 0.30 b	4.00 ± 1.73 ab	15.63 ± 0.54 ab
HDP-B	21.36 ± 0.91 c	78.63 ± 0.91 a	16.30 ± 1.70 b	3.00 ± 1.00 b	14.54 ± 1.63 b

Mean ± standard deviation (*n* = 3). Means from the same column followed by different letters indicate significant differences in the function of the treatment (*p* < 0.05) by one-way ANOVA followed by Tukey’s HSD test. Abbreviations: CTR, control burger; LDP-B, low-fat beef burger formulated with inulin LDP; HDP-B, low-fat beef burger formulated with inulin HDP.

## Data Availability

The original contributions presented in the study are included in the article, further inquiries can be directed to the corresponding author.

## Abbreviations

The following abbreviations are used in this manuscript:

LDP	Low Degree of Polymerization
HDP	High Degree of Polymerization
CTR	Control burger
LDP-B	Low-fat beef burger formulated with inulin LDP
HDP-B	Low-fat beef burger formulated with inulin HDP
DP	Degree of Polymerization
